# Effects of Tributyrin on Antioxidant Capacity, Immune Function, and Liver Macrophage Polarization in Weaned Piglets Under LPS Challenge

**DOI:** 10.3390/ani15192842

**Published:** 2025-09-29

**Authors:** Meng Yuan, Shuai Ning, Dongming Yu, Fei Long, Weite Li, Jun Qi, Yaxu Liang, Changming Hong, Yingzhang Tang, Chunxue Liu, Gaiqin Wang, Bencheng Wu, Xiang Zhong

**Affiliations:** 1 College of Animal Science and Technology, Nanjing Agricultural University, Nanjing 210095, China; yuanmeng@stu.njau.edu.cn (M.Y.); 2023805168@stu.njau.edu.cn (S.N.); 2022205042@stu.njau.edu.cn (D.Y.); longfei@stu.njau.edu.cn (F.L.); liweite@stu.njau.edu.cn (W.L.); qijun@stu.njau.edu.cn (J.Q.); liangyaxu@stu.njau.edu.cn (Y.L.); hongchangming@stu.njau.edu.cn (C.H.); tangyingzhang@stu.njau.edu.cn (Y.T.); liuchunxue@stu.njau.edu.cn (C.L.); 2 Institute of Animal Nutrition and Health Industry, Anyou Biotechnology, Nanjing Agricultural University, Nanjing 210095, China; wanggaiqin@163.com (G.W.); wubencheng@163.com (B.W.); 3 Natural Plant and Animal Health Innovation Institute, NJAU-Cohoo Biotechnology, Nanjing Agricultural University, Nanjing 210095, China

**Keywords:** antioxidant, immunity, liver, macrophage polarization, serum, tributyrin, weaned piglets

## Abstract

**Simple Summary:**

Due to underdeveloped organ systems and inadequate acquisition of maternal antibodies, early-weaned piglets exhibit increased susceptibility to stress, often resulting in reduced growth rates and elevated mortality. This study established a stress model in weaned piglets using LPS and investigated the effects of tributyrin on antioxidant capacity, immune function, and macrophage polarization, along with the underlying mechanisms involved. The results revealed that dietary supplementation with tributyrin significantly enhanced the growth performance of weaned piglets. Moreover, this study showed that under LPS challenge, tributyrin inhibits the activation of the NF-κB signaling pathway via upregulation of SIRT1, while concurrently activating the JAK2/STAT6 signaling pathway. This promotes macrophage polarization from the M1 to the M2 phenotype, thereby enhancing antioxidant capacity and immune function in weaned piglets, attenuating the onset and progression of liver inflammation, and ultimately supporting overall animal health and growth.

**Abstract:**

Under intensive farming systems and the global ban on antibiotic growth promoters (AGPs), early-weaned piglets exhibit incomplete physiological development, increasing their susceptibility to stress-related liver dysfunction and growth performance impairments. This study first investigated the effects of dietary supplementation with 0.2% tributyrin on the growth performance of 21-day-old weaned piglets over a 28-day period. Subsequently, on the final day, we examined its influence on antioxidant capacity, immune responses, and liver macrophage polarization using a 2 × 2 factorial challenge model, with the factors being diet (basal or tributyrin-supplemented) and immunological challenge (saline or lipopolysaccharide). The experimental results indicated that tributyrin had a significant enhancement on the average daily gain (ADG) of weaned piglets within the 0–14-day period (*p* < 0.05). Under lipopolysaccharide (LPS) challenge, tributyrin significantly increased the levels of catalase (CAT) and interleukin-10 (IL-10) while reducing the levels of malondialdehyde (MDA) and interleukin-6 (IL-6) in both serum and liver. Additionally, it significantly increased glutathione peroxidase (GSH-pX) activity in the serum and reduced glutathione (GSH) levels in the liver, and also decreased the serum level of interleukin-1β (IL-1β). Tributyrin downregulated pro-inflammatory cytokine gene expression while upregulating anti-inflammatory cytokine expression (*p* < 0.05). Furthermore, tributyrin significantly inhibited the expression of M1 macrophage polarization markers while enhancing those of M2 polarization (*p* < 0.05). Additionally, tributyrin suppressed SIRT1/NF-κB signaling pathway activation and promoted JAK2/STAT6 signaling pathway activation (*p* < 0.05). These findings exhibit that tributyrin alters the polarization of liver macrophages by regulating the SIRT1/NF-κB and JAK2/STAT6 signaling pathways, enhances antioxidant and immune functions, reduces LPS-induced liver inflammatory damage, and improves the growth performance of weaned piglets.

## 1. Introduction

Global demand for pork, a cost-effective protein source, has grown rapidly with rising living standards, driving the expansion of swine production [1]. In swine production, early weaning is widely adopted to enhance sow reproductive efficiency. At weaning, piglets experience physiological challenges characterized by incomplete organogenesis and abrupt termination of maternal antibody transfer, resulting in compromised immunological competence and increased disease susceptibility [2]. In intensive farming systems, various stressors, including environmental challenges, physiological changes, and nutritional imbalances, frequently trigger stress responses [3]. Such stress can disrupt immune and metabolic functions, potentially causing liver damage that leads to growth retardation and increased mortality. While dietary antibiotic supplementation was proven effective in mitigating such inflammatory responses and organ damage [4], the escalating issue of antimicrobial resistance (AMR) due to antibiotic overuse has prompted worldwide bans on antibiotic growth promoters (AGPs) [5]. Consequently, identifying natural antibiotic alternatives to address post-weaning developmental deficits and stress-induced pathologies in piglets has become a critical research focus in animal husbandry.

The liver is a pivotal multi-functional organ in weaned piglets, orchestrating critical roles in nutrient metabolism, detoxification, and immune surveillance—functions that are particularly vital during the post-weaning transition [6]. As the central hub for metabolic homeostasis, it regulates carbohydrate, lipid, and protein metabolism to support rapid growth, while its detoxification system neutralizes exogenous toxins and endogenous metabolites, a capacity that is often immature in early-weaned piglets [7]. The liver harbors a diverse array of immune cells, including Kupffer cells (liver-resident macrophages), dendritic cells, and lymphocytes [8], which collectively form a first-line defense against pathogens translocating from the gut. Stress can cause immune and metabolic dysfunction in the livers of piglets, especially when challenged by environmental or physiological perturbations during weaning. Liver dysfunction in weaned piglets is frequently linked to dysregulated macrophage polarization [9]. M1-polarized macrophages act as pro-inflammatory effectors, eliminating pathogens but exacerbating tissue damage via pro-inflammatory cytokines. In contrast, M2-polarized macrophages exert anti-inflammatory effects and promote tissue repair through anti-inflammatory mediators [10,11]. Lipopolysaccharide (LPS), a key component of Gram-negative bacterial cell walls, acts as a potent trigger of liver inflammation in weaned piglets. Due to immature intestinal barrier function post-weaning, LPS easily translocates across the gut epithelium, enters the portal circulation via the portal vein, accumulates in the liver, and subsequently reaches the systemic circulation. This translocation activates liver immune responses, drives macrophage dysregulation, and leads to subsequent liver injury, characterized by oxidative stress, inflammatory cytokine release, and impaired metabolic function [12,13].

Tributyrin is an esterified form of butyric acid [14,15], one of the derivatives of butyric acid, and shows superior stability, prolonged shelf-life, and enhanced gastric transit capability compared to free butyrate. It is absorbed and utilized in the hindgut or transported to other tissues and organs of the body along with blood circulation. Tributyrin possesses well-documented antioxidant, anti-inflammatory, immunomodulatory, and microbiota-regulating properties [16]. Studies have found that tributyrin can significantly improve the activity of antioxidant enzymes, enhance the secretion of anti-inflammatory cytokines [17], alleviate DSS-induced intestinal inflammation, and reduce intestinal damage [18]. Moreover, tributyrin regulates epigenetic modifications to inhibit the secretion of inflammatory cytokines and increases the abundance of beneficial intestinal flora [19]. Additionally, tributyrin inhibits bacteria from producing toxic metabolites [20], relieves intestinal inflammatory damage, and improves animal growth performance [21]. Current research regarding the effects of tributyrin on animal health remains incomplete, especially regarding its impact on liver function and macrophage polarization in weaned piglets, with underlying mechanisms remaining unclear. Thus, the aim of this study was to investigate the effects of tributyrin on the growth performance of early-weaned piglets and on antioxidant capacity, immune function, and liver macrophage polarization in weaned piglets under LPS challenge.

## 2. Materials and Methods

### 2.1. Experimental Animals and Materials

Weaned piglet experiments were conducted following guidelines authorized by the Animal Ethics Committee of Nanjing Agricultural University (Permit number SYXK-2022-0031). Weaned piglets (Duroc × Landrace × Large White ternary crossbred) were obtained from Taicang Breeding Farm. The tributyrin supplement (purity: 65%) was provided by Shanchuan Biological.

### 2.2. Experimental Design

In this experiment, 21-day-old weaned piglets (Duroc × Landrace × Large White, with equal numbers of males and females, *n* = 72) with similar body weight were used as the experimental animals. The feeding experiment was conducted at Anyou Pig Farm in Taicang. In a completely randomized design (CRD), 72 pigs were randomly assigned to two groups (control group fed with a basal diet: control; tributyrin group fed with a basal diet supplemented with 0.2% tributyrin: tributyrin), each consisting of 6 replicates with 6 pigs per replicate. Each replicate was housed in a separate pen divided by fencing. The experiment consisted of a 3-day adaptation period followed by a 28-day formal trial. Feed intake and fasting body weight (fasted for 8 h) were recorded. On the 28th day of the experiment, two piglets were randomly selected from each replicate of the control and tributyrin groups to undergo the subsequent injection treatments. The piglets were assigned to the following four treatment groups: (1) Negative control (NC): Fed a basal diet and intraperitoneally injected with 80 μg/kg of normal saline. (2) Tributyrin (TB): Fed a basal diet supplemented with 0.2% tributyrin and intraperitoneally injected with 80 μg/kg of normal saline. (3) Negative control LPS treatment (LPS): Fed a basal diet and intraperitoneally injected with 80 μg/kg of LPS (93572-42-0, Sigma-Aldrich, St. Louis, MO, USA). (4) Tributyrin LPS treatment (TL): Fed a basal diet containing 0.2% tributyrin and intraperitoneally injected with 80 μg/kg of LPS. After 4 h of intraperitoneal injection of LPS or normal saline, blood samples were collected from the piglets, and their rectal temperature was measured. Subsequently, the piglets were euthanized for sample collection.

### 2.3. Feeding Management and Diet Composition

The experimental facility was equipped with a slatted-floor system to ensure optimal ventilation and thermal insulation, maintaining a controlled environment throughout the trial. Weaned piglets were provided with ad libitum access to nutritionally balanced pelleted feed and fresh water to meet their physiological requirements. Daily monitoring protocols included systematic morning assessments of feed intake, residual feed quantity, and individual health status (e.g., signs of morbidity, abnormal behavior, or distress). All vaccination procedures were strictly followed by the farm’s standardized biosecurity protocols, while facility sanitation was maintained in accordance with established hygienic operating procedures to minimize cross-contamination risks. Basal diet composition and nutrient levels (NRC 2012) [22] are shown in Table 1.

### 2.4. Sample Collection

Four hours after intraperitoneal injection of LPS or normal saline into piglets, rectal temperatures were measured and recorded using a calibrated digital thermometer (MAODENGKEJI, 32.00~42.99 °C). Blood samples were collected from the anterior vena cava using sterile venipuncture techniques and transferred into procoagulant tubes according to standard protocols. After allowing 2 h of clotting at room temperature (25 °C), samples were centrifuged at 3000× *g* for 10 min (Desktop low-speed centrifuge, CenLee, TD4D). The obtained serum was aliquoted and stored at −20 °C for subsequent biochemical analyses. The blood on the liver surface was washed with normal saline, and the fixed and frozen samples of piglet liver tissue (the same position of the right lobe of the liver) were collected.

### 2.5. Index Determination

#### 2.5.1. Determination of Growth Performance of Weaned Piglets

On the initial day of the formal trial period, piglets were fasted and weighed to establish baseline body weights. Subsequent fortnightly measurements included both body weight determinations and precise feed intake recordings.

#### 2.5.2. Determination of Antioxidant and Immune Indexes 

Serum and liver antioxidant status was evaluated by measuring the following parameters using commercial kits: total superoxide dismutase (T-SOD; Catalogue No. A001-3-2), glutathione peroxidase (GSH-pX; Catalogue No. A005-1-2), reduced glutathione (GSH; Catalogue No. A006-2-1), catalase (CAT; Catalogue No. A007-1-1), and total antioxidant capacity (T-AOC; Catalogue No. A015-2-1) from Nanjing Jiancheng Bioengineering Institute (Nanjing, China). Malondialdehyde levels (MDA; Catalogue No. BC0025) were determined using kits obtained from Beijing Solaibao Biotechnology Co., Ltd. (Beijing, China).

Serum and liver concentrations of pro- and anti-inflammatory cytokines were quantified using ELISA kits: interleukin-1β (IL-1β; Catalogue No. ml002302), interleukin-6 (IL-6; Catalogue No. ml002311), tumor necrosis factor-α (TNF-α; Catalogue No. ml002360), and interleukin-10 (IL-10; Catalogue No. ml002319), all purchased from Shanghai Enzyme-Linked Biotechnology Co., Ltd. (Shanghai, China).

All measurements were performed with strict adherence to the standardized procedures. To allow for comparisons between samples, the obtained data were normalized to the total protein content of each sample.

#### 2.5.3. Determination of Serum Biochemical Indices

Serum aspartate aminotransferase (AST; Catalogue No. C010-2-1) and alanine aminotransferase (ALT; Catalogue No. C009-2-1) activities were determined using commercial assay kits (Nanjing Jiancheng Bioengineering Institute, Nanjing, China) according to the manufacturer’s protocols. All measurements were performed with strict adherence to the standardized procedures.

#### 2.5.4. mRNA Expression Analysis

Total RNA was extracted from the liver using RNA extraction reagent (AG RNAex Pro Reagent, No. AG21102, Accurate Biotechnology (Changsha, China)) according to the protocol from the manufacturer. RNA concentration and purity were determined spectrophotometrically (A260/A280 ratio > 1.8, A260/A230 > 2.0). The RNA concentration determined was calculated and adjusted to 500 ng/μL using RNA-free water. Using the reverse transcription kit (Evo M-MLV RT Mix Kit with gDNA Clean for qPCR Ver. 2, No. AG11728, Accurate Biotechnology), the RNA was reverse-transcribed into cDNA, and then 180 μL of nuclease-free water was added for mixing. Subsequently, the reaction system was assembled using the qPCR detection kit (SYBR Green Pro Taq HS qPCR Kit (Rox Plus), No. AG11718, Accurate Biotechnology), and qPCR was performed. 

The mRNA expression levels of cytokines (IL-1β, TNF-α, IL-6, IL-4, IL-10, IL-13), macrophage polarization-related markers (iNOS, CD86, Arg1, CD206), and key genes in the SIRT1/NF-κB and JAK2/STAT6 signaling pathways were detected by quantitative real-time PCR. *β-actin* was used as the internal reference, and all primer sequences were designed using the NCBI database. The relative expression levels of genes were calculated by the 2^−ΔΔCt^ method. The primer sequences are shown in Table 2.

The reaction system is as follows: each 10 μL reaction contained 2 μL cDNA template, 0.2 μL forward/reverse primers, 5 μL 2 × SYBR Green Pro Taq HS Premix, 2.6 μL nuclease-free water. The amplification protocol consisted of three sequential steps: (1) initial denaturation at 95 °C for 5 min to activate the DNA polymerase; (2) 40 amplification cycles comprising denaturation at 95 °C for 10 s followed by primer annealing/extension at 60 °C for 30 s; (3) a final melting curve analysis with temperature ramping from 60 °C to 95 °C (15 s at each temperature endpoint).

#### 2.5.5. Immunofluorescence (IF)

Following standard tissue fixation and paraffin embedding procedures, serial sections were processed for immunofluorescence analysis. The sections underwent sequential treatment, including baking for tissue adhesion, dewaxing in xylene, 100%, 95%, 85%, and 75% ethanol solution, and were then placed in a container filled with antigen retrieval buffer (Cat. No. P0088, Biyuntian Biotechnology, Nanjing, China) and boiled for 10 min. Then, the sealing liquid (Cat. No. P0260, Biyuntian Biotechnology, Nanjing, China) was applied for sealing for 1 h. After adding the primary antibodies CD206 and F4/80, the sections were placed in the refrigerator at 4 °C for incubation for 12 h and then washed with PBS three times for 5 min each time. Subsequently, the secondary antibody was added and incubated at room temperature for 40 min, after which the sections were washed with PBS three times for 5 min each time. From this point onward, all steps were performed under light-protected conditions. Finally, the DAPI staining solution was added and incubated for 15 min, followed by washing with PBS three times for 5 min each time. Immunofluorescence visualization was conducted using a Zeiss LSM710 confocal laser scanning microscope (Carl Zeiss AG, Jena, Germany) with consistent imaging parameters. Captured images were subsequently quantified for fluorescence intensity using ImageJ software (ImageJ 1.54d, NIH, USA), with complete antibody specifications detailed in Table 3.

#### 2.5.6. Western Blot 

Frozen liver tissue samples were retrieved from −80 °C storage and homogenized in RIPA lysis buffer (containing 1 mM PMSF and 50× phosphatase inhibitor) using a grinding tube. The homogenate was centrifuged at 12,000× *g* for 10 min at 4 °C, and the supernatant was collected for protein quantification. Protein concentration was determined using a BCA assay kit (Cat. No. P0010, Biyuntian Biotechnology, Nanjing, China) according to the manufacturer’s protocol, with samples normalized to a uniform concentration of 4 μg/μL. Protein samples were mixed with 2× SDS-PAGE loading buffer (1:1, *v*/*v*), denatured at 95 °C for 10 min, and briefly centrifuged. Equal amounts of protein (10 μL per lane) were separated by SDS-PAGE on 4–20% gels at 140 V for 1 h, followed by electrophoretic transfer to PVDF membranes (300 mA, 45 min). Membranes were blocked with 5% skim milk in TBST for 2 h at room temperature with gentle agitation. After blocking, membranes were incubated overnight at 4 °C with primary antibodies (dilutions and sources listed in Table 3), washed 3 × 10 min with TBST, and then probed with horseradish peroxidase (HRP)-conjugated secondary antibodies for 1.5 h at room temperature. Following three additional TBST washes (10 min each), protein bands were detected using enhanced chemiluminescence (ECL) and imaged with a chemiluminescent documentation system (Fujifilm Co., Ltd., Tokyo, Japan). Band intensities were quantified using ImageJ software (National Institutes of Health, Bethesda, MD, USA).

### 2.6. Data Statistics and Analysis

The experimental data were subjected to normal distribution and homogeneity of variance tests. Growth performance data were analyzed using two-tailed Student’s *t*-tests. The remaining data were analyzed by two-way analysis of variance (ANOVA) using SPSS 26.0 software (IBM Corp., Armonk, NY, USA). The experiment included two main effects: tributyrin treatment and LPS challenge, with their interaction effect representing the combined impact of tributyrin treatment and LPS challenge. Post-hoc comparisons were adjusted using Tukey’s test for multiple comparisons. The results were expressed as “Mean± standard error of the mean” (Mean ± SEM) and plotted using GraphPad Prism 9.0 software. *p* < 0.05 indicates a significant statistical difference.

## 3. Results

### 3.1. Effects of Tributyrin on the Growth Performance of Weaned Piglets

Table 4 shows the effect of tributyrin on the growth performance of weaned piglets. During the initial 14-day period, tributyrin supplementation significantly enhanced the average daily gain (ADG, *p* < 0.05) of piglets, tended to improve average daily feed intake (ADFI, *p* = 0.087), but did not affect feed efficiency (G:F, *p* > 0.05). In the subsequent period (days 15–28), although tributyrin had no significant impact on growth performance parameters, including ADG, ADFI, and G:F (*p* > 0.05), tributyrin supplementation numerically improved growth performance in weaned piglets, increasing average daily gain (ADG) by 5.57% and average daily feed intake (ADFI) by 3.11%. Comprehensive analysis of the entire 28-day experimental period showed that although tributyrin had no significant impact on overall growth performance parameters, including ADG, ADFI, and G:F (*p* > 0.05), tributyrin supplementation numerically improved growth performance in weaned piglets, increasing average daily gain (ADG) by 4.31% and average daily feed intake (ADFI) by 4.25%. The results showed that tributyrin could enhance the early growth performance of weaned piglets.

### 3.2. The Effects of Tributyrin on Antioxidant Function in Serum and Liver of Weaned Piglets Under LPS Challenge

**Figure 1:**
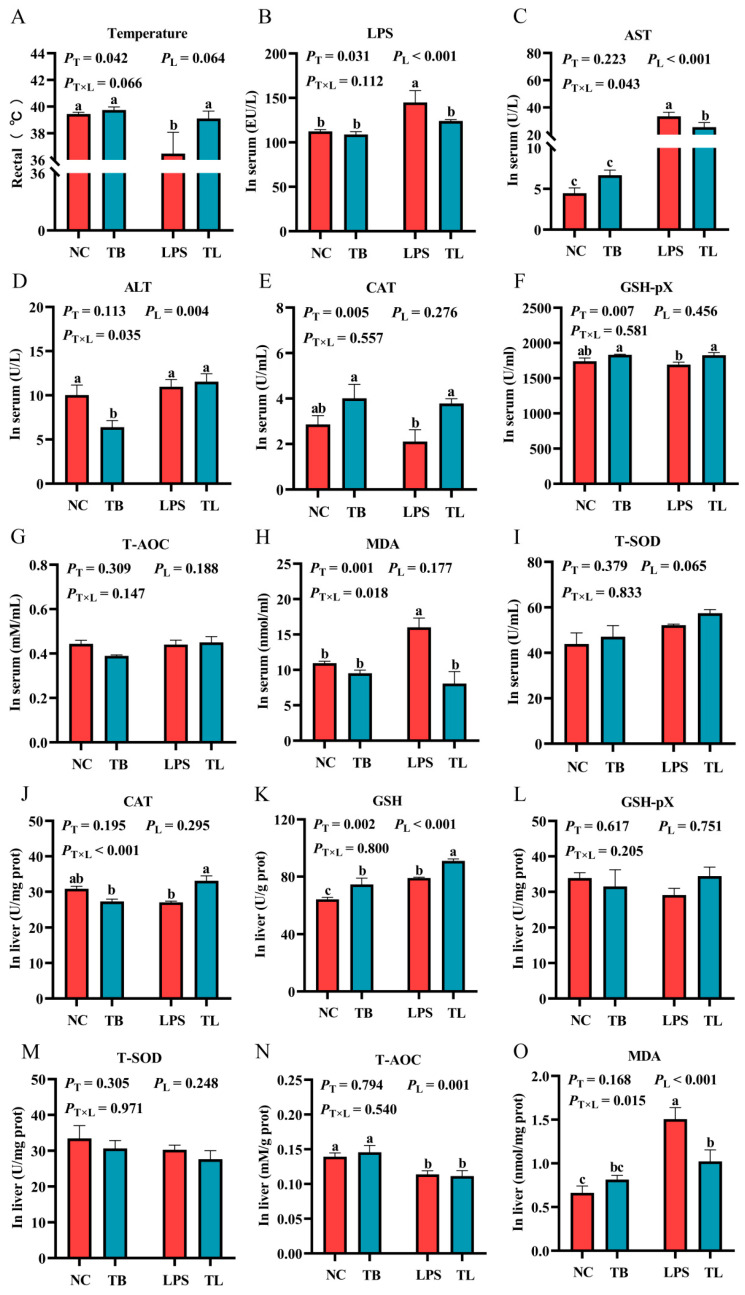
The effects of tributyrin on antioxidant function in serum and liver of weaned piglets under LPS challenge. (**A**) The rectal temperature of weaned piglets under LPS challenge. (**B**) The content of LPS in the serum of weaned piglets under LPS challenge. (**C**,**D**) The content of AST and ALT in the serum of weaned piglets under LPS challenge, respectively. (**E**–**I**) The antioxidant enzyme activities and oxidative damage indicators in the serum of weaned piglets under LPS challenge. (**J**–**O**) The antioxidant enzyme activities and oxidative damage indicators in the liver of weaned piglets under LPS challenge. Values with the same lowercase letters indicate no significant difference (*p* > 0.05), and different lowercase letters indicate significant differences (*p* < 0.05). *P*_T_: The main effect *p*-value of tributyrin, *P*_L_: The main effect *p*-value of LPS, *P*_T×L_: The interaction effect *p*-value of tributyrin and LPS, and *p* < 0.05 indicates a significant difference. Negative control (NC): Fed with a basal diet and 80 μg/kg of normal saline was intraperitoneally injected, Tributyrin (TB): Fed with a basal diet containing 0.2% tributyrin and 80 μg/kg of normal saline was intraperitoneally injected, Negative control LPS treatment (LPS): Fed with a basal diet and 80 μg/kg of LPS was intraperitoneally injected, Tributyrin LPS treatment (TL): Fed with a basal diet containing 0.2% tributyrin and 80 μg/kg of LPS was intraperitoneally injected. T-SOD: Total superoxide dismutase, CAT: Catalase, T-AOC: Total antioxidant, GSH-pX: Glutathione peroxidase, GSH: Reduced glutathione, MDA: Malondialdehyde.

To investigate the effects of LPS and tributyrin on the antioxidant function of weaned piglets under LPS challenge, we first measured the rectal temperature and serum LPS levels in weaned piglets (Figure 1A,B). Compared with the LPS group, tributyrin could significantly inhibit the decrease in body temperature caused by LPS and protect weaned piglets from the damage caused by body temperature imbalance (Figure 1A). Results revealed that the interaction effect between tributyrin and LPS challenge was not significant (*p* = 0.066) but showed a statistical trend. Both tributyrin and LPS challenge significantly influenced serum LPS levels in weaned piglets (*p* < 0.05). Intraperitoneal LPS challenge substantially increased circulating LPS concentrations. Together with the corresponding changes in body temperature, these results showed the successful induction of an LPS-mediated stress model. Furthermore, this study found that tributyrin effectively attenuated the LPS content in the serum under LPS challenge.

Then, to explore whether tributyrin can alleviate liver injury in stress-weaned piglets induced by LPS, the contents of liver injury indices aspartate aminotransferase (AST) and alanine aminotransferase (ALT) in serum were measured. Compared with the NC group, the LPS group significantly increased the content of aspartate aminotransferase (AST) in serum (*p* < 0.05) (Figure 1C), and tributyrin was able to significantly reduce the content of alanine aminotransferase (ALT) (*p* < 0.05) (Figure 1D). Under LPS challenge, tributyrin significantly decreased the content of aspartate aminotransferase (AST) in serum compared with the LPS group (*p* < 0.05) (Figure 1C). A significant interaction was observed, whereby serum AST levels were reduced by tributyrin (TB) exclusively under LPS challenge, while serum ALT levels were reduced only in the absence of LPS challenge. Data showed that dietary supplementation with tributyrin effectively alleviated liver damage caused by LPS.

In order to explore the effect of tributyrin on the antioxidant function of LPS-stressed weaned piglets, we determined the antioxidant activity and oxidative damage indicators in the liver and serum. Compared with the NC group, the content of malondialdehyde (MDA) in the serum was significantly increased in the LPS group (*p* < 0.05). Under LPS challenge, tributyrin significantly increased the activities of catalase (CAT) and glutathione peroxidase (GSH-pX) (*p* < 0.05) while decreasing malondialdehyde (MDA) content in the serum (*p* < 0.05) (Figure 1E,F,H). Moreover, the significant interaction between tributyrin and LPS challenge on malondialdehyde (MDA) content (*p* < 0.05) (Figure 1H) in the serum was indicated by a significant reduction in malondialdehyde (MDA) levels induced by tributyrin solely in the presence of LPS challenge. Likewise, compared with the NC group, total antioxidant capacity (T-AOC) in the liver was significantly decreased, and the content of malondialdehyde (MDA) and reduced glutathione (GSH) in the liver was significantly increased in the LPS group (*p* < 0.05) (Figure 1K,N,O). Compared with the LPS group, the TL group showed significantly increased activity of catalase (CAT) and content of reduced glutathione (GSH) in the liver (*p* < 0.05), along with significantly decreased malondialdehyde (MDA) content in the liver (*p* < 0.05) (Figure 1J,K,O). Furthermore, a significant interaction between tributyrin and LPS challenge for both catalase (CAT) activity and malondialdehyde (MDA) content (*p* < 0.05) (Figure 1J,O) in the liver was observed, which was shown by a reduction in malondialdehyde (MDA) and an increase in catalase (CAT) activity induced by tributyrin specifically under LPS challenge. Tributyrin improved antioxidant capacity in LPS-challenged weaned piglets.

### 3.3. The Effect of Tributyrin on Immune Function of Weaned Piglets Under LPS Challenge

**Figure 2:**
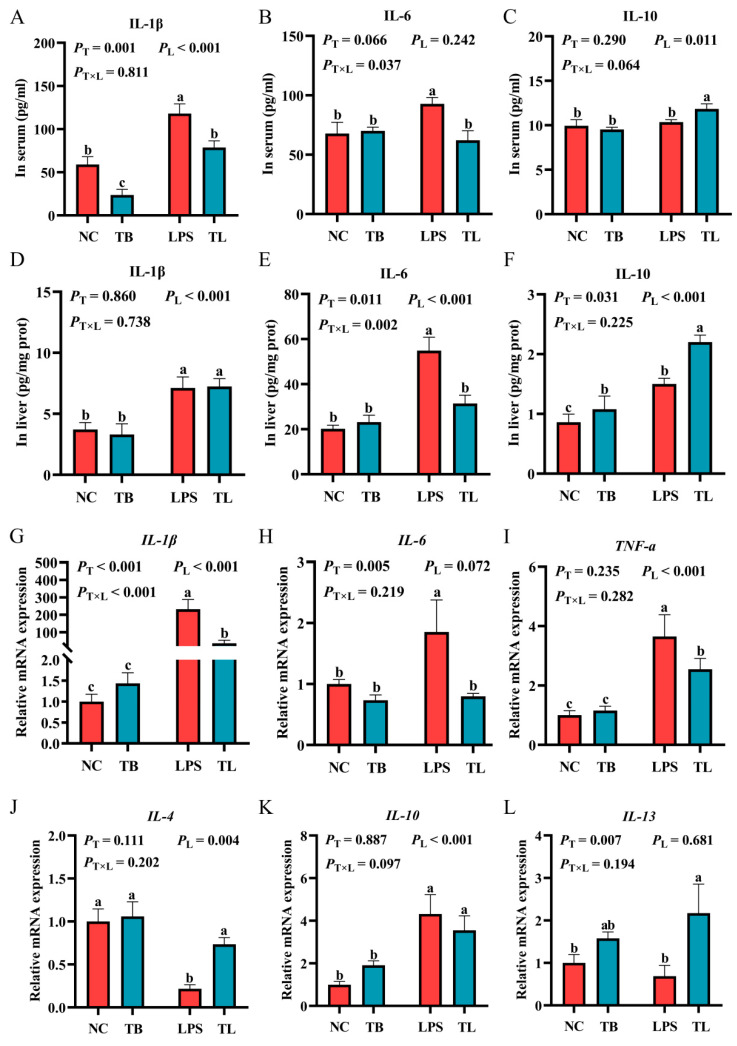
The effect of tributyrin on immune function of weaned piglets under LPS challenge. (**A**–**C**) The cytokine contents in the serum of weaned piglets under LPS challenge. (**D**–**F**) The cytokine contents in the liver of weaned piglets under LPS challenge. (**G**–**L**) Relative mRNA expression levels of *IL-1β*, *IL-6*, *TNF-α*, *IL-4*, *IL-10*, and *IL-13* in the liver. Values with the same lowercase letters indicate no significant difference (*p* > 0.05), and different lowercase letters indicate significant differences (*p* < 0.05). *P*_T_: The main effect *p*-value of tributyrin, *P*_L_: The main effect *p*-value of LPS, *P*_T×L_: The interaction effect *p*-value of tributyrin and LPS, and *p* < 0.05 indicates a significant difference. Negative control (NC): Fed with a basal diet and 80 μg/kg of normal saline was intraperitoneally injected, Tributyrin (TB): Fed with a basal diet containing 0.2% tributyrin and 80 μg/kg of normal saline was intraperitoneally injected, Negative control LPS treatment (LPS): Fed with a basal diet and 80 μg/kg of LPS was intraperitoneally injected, Tributyrin LPS treatment (TL): Fed with a basal diet containing 0.2% tributyrin and 80 μg/kg of LPS was intraperitoneally injected. *IL*: interleukin; *TNF-a*: tumor necrosis factor-α.

To explore the effect of tributyrin on the immune function of weaned piglets under LPS challenge, we determined the contents of inflammatory cytokines and anti-inflammatory cytokines in the liver and serum, and the gene expression of cytokines in liver tissues. Compared with the NC group, the contents of IL-1β and IL-6 in the serum were significantly increased by intraperitoneal injection of LPS (*p* < 0.05) (Figure 2A,B). The TB group significantly reduced the content of IL-1β compared with the NC group (Figure 2A). Under LPS challenge, tributyrin could significantly reduce the contents of IL-1β and IL-6 in the serum (*p* < 0.05) and significantly increase the content of IL-10 (*p* < 0.05) (Figure 2A–C). In addition, a significant interaction between tributyrin and LPS challenge was observed for IL-6 content (*p* < 0.05) (Figure 2B) in the serum, which was shown by a significant reduction in IL-6 by tributyrin specifically under LPS challenge conditions. Similarly, compared with the NC group, the LPS group significantly increased the contents of IL-1β, IL-6, and IL-10 in the liver (*p* < 0.05) (Figure 2D–F). Tributyrin significantly increased the content of IL-10 in the liver (*p* < 0.05) (Figure 2F). Under LPS challenge, tributyrin significantly reduced the content of IL-6 (*p* < 0.05) and significantly increased the content of IL-10 (*p* < 0.05) (Figure 2E,F). Similar to the serum, the level of IL-6 in the liver was only significantly reduced by tributyrin under LPS challenge, and there was no significant change without LPS challenge (*p* < 0.05) (Figure 2E).

In order to explore the effect of tributyrin on the expression of cytokine genes in the liver of weaned piglets under challenge, the relative expression levels of cytokine mRNA in liver tissues were determined by quantitative real-time PCR. When feeding the basal diet, LPS challenge significantly increased the mRNA expression levels of *IL-1β*, *TNF-a*, *IL-6*, and *IL-10* (*p* < 0.05), and significantly decreased the mRNA expression levels of *IL-4* and *IL-13* (*p* < 0.05) (Figure 2G–L). Under LPS challenge, tributyrin significantly decreased the mRNA expression levels of *IL-1β*, *TNF-a*, and *IL-6* (*p* < 0.05) (Figure 2G–I); significantly increased the mRNA expression levels of *IL-4* and *IL-13* (*p* < 0.05) (Figure 2J,L); and there was no significant difference in the mRNA expression level of *IL-10* (*p* > 0.05) (Figure 2K). The significant interaction between tributyrin supplementation and LPS challenge showed that tributyrin reduced *IL-1β* gene expression in the liver exclusively under LPS challenge (*p* < 0.05) (Figure 2G). Tributyrin improved immune responses in LPS-exposed weaned piglets.

### 3.4. The Effect of Tributyrin on the Polarization and Infiltration of Macrophages in the Liver of Weaned Piglets Under LPS Challenge

**Figure 3:**
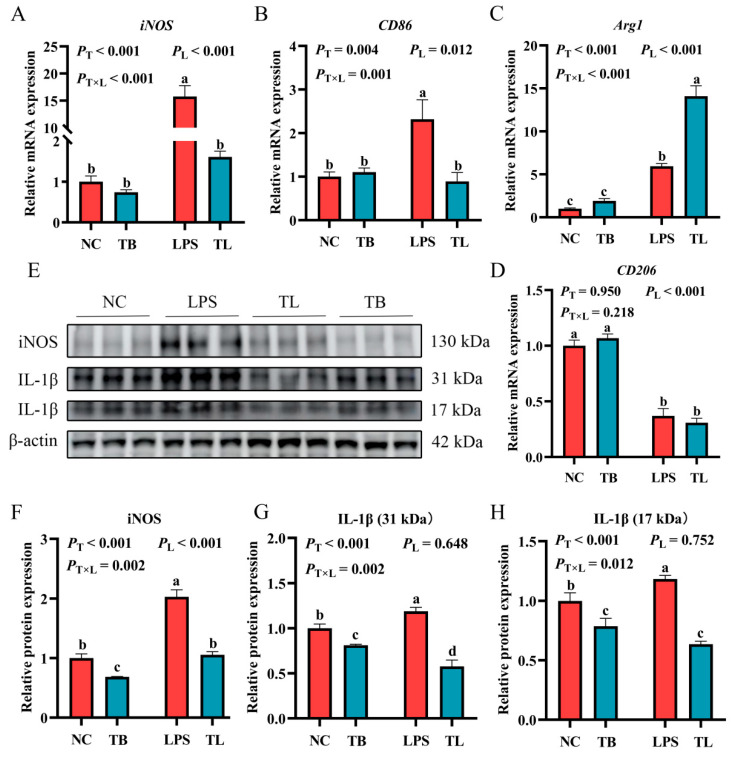
Effects of tributyrin on macrophage marker gene and protein expression in liver of weaned piglets under LPS challenge. (**A**–**D**) The relative gene expression levels of M1 macrophage markers *iNOS* and *CD86* and M2 macrophage markers *Arg1* and *CD206*, respectively. (**E**) Protein expression of iNOS and IL-1β (17 kDa and 31 kDa). (**F**–**H**) The relative protein expression levels of iNOS and IL-1β (17 kDa and 31 kDa), respectively. Values with the same lowercase letters indicate no significant difference (*p* > 0.05), and different lowercase letters indicate significant differences (*p* < 0.05). *P*_T_: The main effect *p*-value of tributyrin, *P*_L_: The main effect *p*-value of LPS, *P*_T×L_: The interaction effect *p*-value of tributyrin and LPS, and *p* < 0.05 indicates a significant difference. Negative control (NC): Fed with a basal diet and 80 μg/kg of normal saline was intraperitoneally injected, Tributyrin (TB): Fed with a basal diet containing 0.2% tributyrin and 80 μg/kg of normal saline was intraperitoneally injected, Negative control LPS treatment (LPS): Fed with a basal diet and 80 μg/kg of LPS was intraperitoneally injected, Tributyrin LPS treatment (TL): Fed with a basal diet containing 0.2% tributyrin and 80 μg/kg of LPS was intraperitoneally injected.

**Figure 4:**
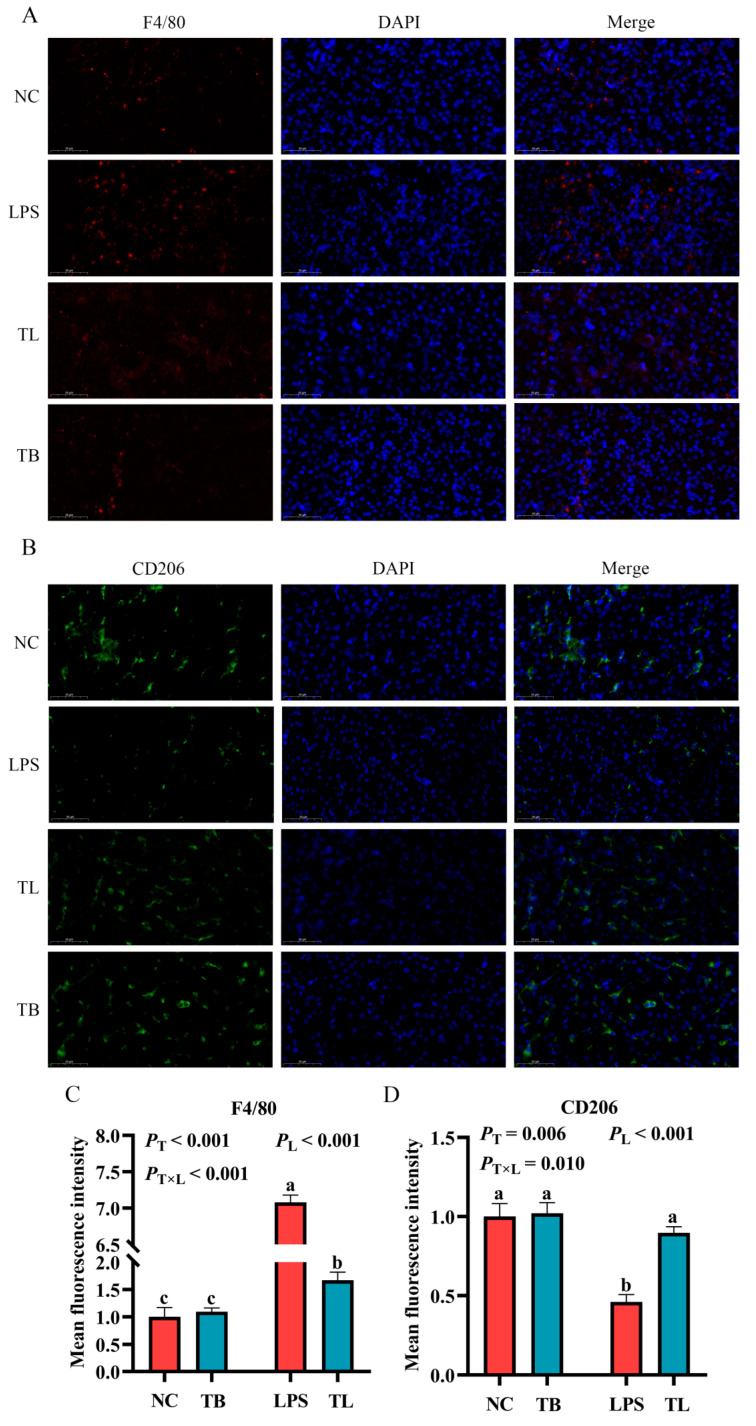
Effects of tributyrin on macrophage infiltration and CD206+ polarization in the liver of weaned piglets under LPS challenge. (**A**,**B**) Representative immunofluorescence images of F4/80 and CD206 are shown at 60× magnification (scale bar = 50 μm). (**C**,**D**) Quantify the positive staining rates of F4/80 and CD206 in piglet liver tissues, respectively. Values with the same lowercase letters indicate no significant difference (*p* > 0.05), and different lowercase letters indicate significant differences (*p* < 0.05). *P*_T_: The main effect *p*-value of tributyrin, *P*_L_: The main effect *p*-value of LPS, *P*_T×L_: The interaction effect *p*-value of tributyrin and LPS, and *p* < 0.05 indicates a significant difference. Negative control (NC): Fed with a basal diet and 80 μg/kg of normal saline was intraperitoneally injected, Tributyrin (TB): Fed with a basal diet containing 0.2% tributyrin and 80 μg/kg of normal saline was intraperitoneally injected, Negative control LPS treatment (LPS): Fed with a basal diet and 80 μg/kg of LPS was intraperitoneally injected, Tributyrin LPS treatment (TL): Fed with a basal diet containing 0.2% tributyrin and 80 μg/kg of LPS was intraperitoneally injected.

The above data indicate that LPS challenge can cause the imbalance of antioxidant and immune homeostasis and cause an inflammatory response in weaned piglets, and dietary tributyrin supplementation can effectively reverse these results. The occurrence and progression of inflammation are often accompanied by the infiltration of a large number of macrophages and changes in macrophage function [23]. Therefore, in order to explore the effects of tributyrin on macrophages in weaned piglets with LPS challenge, quantitative real-time PCR (qRT-PCR), Western blot, and immunofluorescence techniques were used to determine the gene and protein expression of macrophage markers in the liver. When feeding the basal diet, the mRNA expression levels of *CD86* and *iNOS* were significantly increased by LPS (*p* < 0.05), while the mRNA expression level of *CD206* was significantly decreased (*p* < 0.05) (Figure 3A,B,D). Under LPS challenge, tributyrin significantly increased the mRNA expression level of *Arg1* (*p* < 0.05) (Figure 3C). Furthermore, tributyrin and LPS challenge interacted significantly to modulate the gene expression of *iNOS*, *CD86*, and *Arg1* in the liver (*p* < 0.05) (Figure 3A–C). The expression of the M1 markers *iNOS* and *CD86* was significantly suppressed by tributyrin only under LPS challenge. Conversely, tributyrin significantly enhanced the expression of the M2 marker *Arg1*, also specifically under LPS challenge. Additionally, compared with the NC group, the protein expressions of iNOS and IL-1β were significantly increased by LPS (*p* < 0.05) (Figure 3F–H). Under the challenge of LPS, the protein expression of iNOS and IL-1β was significantly increased (*p* < 0.05), but tributyrin had significant reductions in the protein expression of iNOS and IL-1β (*p* < 0.05) (Figure 3F–H). More importantly, tributyrin significantly reduced the protein expression levels of both iNOS and IL-1β (17kDa and 31kDa), irrespective of LPS challenge (*p* < 0.05) (Figure 3F–H). LPS challenge significantly increased the positive rate of F4/80 compared with the NC group (*p* < 0.05) (Figure 4A,C). Under LPS challenge, the CD206 positive rate was significantly increased by tributyrin (*p* < 0.05), while the F4/80 positive rate was significantly decreased (*p* < 0.05) (Figure 4B,D). The positive rate of F4/80 was significantly reduced by tributyrin only under LPS challenge. Conversely, the positive rate of CD206 was significantly enhanced by tributyrin specifically under LPS challenge (*p* < 0.05) (Figure 4A–D). The above data indicated that tributyrin reduced macrophage infiltration in the liver, inhibited M1 polarization, and promoted M2 polarization.

### 3.5. Effects of Tributyrin on the SIRT1/NF-κB and JAK2/STAT6 Signaling Pathways in the Liver of LPS Stress-Induced Weaned Piglets

**Figure 5:**
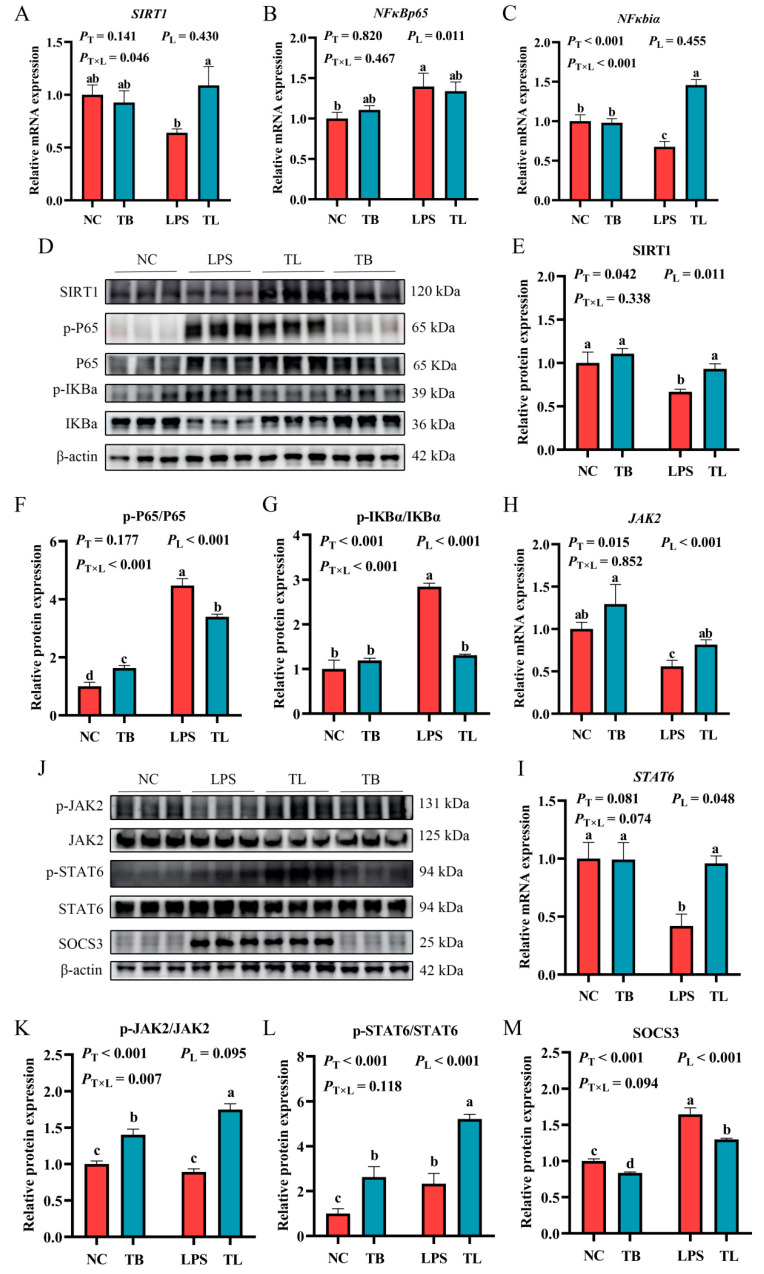
Effect of tributyrin on SIRT1/NF-κB and JAK2/STAT6 signaling pathways in liver of LPS-stressed weanling piglets. (**A**–**C**) The relative gene expression levels of *SIRT1*, *NFκBp65*, and *NFκbiα* in the SIRT1/NF-κB signaling pathway. (**H**–**I**) The relative gene expression levels of *JAK2* and *STAT6* in the JAK2/STAT6 signaling pathway. (**D**–**J**) The protein expression levels of key proteins in the SIRT1/NFκB and JAK2/STAT6 signaling pathways. (**E**–**G**,**K**–**M**) The relative expression levels of proteins in the pathways. Values with the same lowercase letters indicate no significant difference (*p* > 0.05), and different lowercase letters indicate significant differences (*p* < 0.05). *P*_T_: The main effect *p*-value of tributyrin, *P*_L_: The main effect *p*-value of LPS, *P*_T×L_: The interaction effect *p*-value of tributyrin and LPS, and *p* < 0.05 indicates a significant difference. Negative control (NC): Fed with a basal diet and 80 μg/kg of normal saline was intraperitoneally injected, Tributyrin (TB): Fed with a basal diet containing 0.2% tributyrin and 80 μg/kg of normal saline was intraperitoneally injected, Negative control LPS treatment (LPS): Fed with a basal diet and 80 μg/kg of LPS was intraperitoneally injected, Tributyrin LPS treatment (TL): Fed with a basal diet containing 0.2% tributyrin and 80 μg/kg of LPS was intraperitoneally injected.

Finally, in order to explore the molecular mechanism of the effects of tributyrin on inflammation and macrophage polarization, the expression levels of key genes and proteins in the SIRT1/NF-κB and JAK2/STAT6 signaling pathways were determined by quantitative real-time PCR and Western blot. In the SIRT1/NF-κB signaling pathway, compared with the NC group, the LPS group decreased the mRNA expression levels of *SIRT1* and *NFκbiα* (*p* < 0.05), and significantly increased the mRNA expression level of *NFκBp65* (*p* < 0.05) (Figure 5A–C). Meanwhile, the relative protein expression levels of p-P65/P65 and p-IKBα/IKBα were significantly increased (*p* < 0.05), and the relative protein expression level of SIRT1 was significantly decreased (*p* < 0.05) (Figure 5E–G). Specifically, tributyrin significantly increased the gene expression of *SIRT1* and *NFκBiα* while reducing the phosphorylation of NFκBp65 and IκBα, an effect that was specific to the presence of LPS challenge (*p* < 0.05) (Figure 5A–C). In the JAK2/STAT6 signaling pathway, compared with the NC group, LPS group significantly reduced the mRNA expression levels of JAK2 and STAT6 (*p* < 0.05), the relative protein expression level of SOCS3 was significantly increased (*p* < 0.05), while tributyrin could significantly reverse these results (Figure 5H–M). It was also found that LPS challenge increased the relative protein expression level of p-STAT6/STAT6 (Figure 5L), possibly due to adaptive regulation of macrophage polarization homeostasis during M1 polarization. Moreover, the relative protein expression level of p-STAT6/STAT6 was highly significantly increased by the tributyrin (*p* < 0.05). Additionally, the phosphorylation level of JAK2 was significantly increased by tributyrin, irrespective of LPS challenge (*p* < 0.05). The above data indicate that tributyrin can inhibit the activation of inflammatory signaling pathways and M1 polarization of macrophages and promote M2 polarization of macrophages by inhibiting the activation of the SIRT1/NF-κB signaling pathway and promoting the activation of the JAK2/STAT6 signaling pathway.

## 4. Discussion

In the intensive breeding model, due to the existence of various stressors such as insufficient nutrient intake, immunization, and environmental factors, early weaned piglets are more prone to stress responses, resulting in stunted growth and development of piglets in the early stage, and even death [24]. Studies have shown that adding tributyrin to the diet can improve the growth performance of animals [25,26]. In the study of piglets, the authors found that dietary supplementation with tributyrin could enhance the early growth performance of weaned piglets and reduce the proportion of piglets with growth retardation [27]. Our experiment was consistent with the results of previous studies: adding 0.2% tributyrin to the diet can significantly improve the early growth performance of weaned piglets and promote their growth and development. The above results indicate that dietary supplementation with tributyrin can promote the growth and development of animals. This could be because tributyrin is decomposed into butyric acid in the hindgut and is then transported into the blood by the monocarboxylic acid transporter (MCT1), reaching the liver and other tissues via the portal vein. In addition, it participates in the β-oxidation of tissue sites to generate Acetyl-CoA, enters the tricarboxylic acid cycle (TCA), provides energy for cell proliferation and growth, and thereby promotes their growth and development [28].

Oxidative stress and immune stress can cause severe damage to the organs of livestock and poultry in commercial production and are regarded as one of the factors restricting the growth of piglets. Therefore, enhancing the antioxidant and immune capabilities of animals is also one of the effective measures to resist stress responses. Previous studies have found that adding tributyrin to the diet can enhance the serum antioxidant capacity of yellow-feathered broilers, improve the development of the gut, regulate the composition of the gut microbiota, and improve the growth of broilers [29]. Intraperitoneal administration of lipopolysaccharide (LPS) triggers acute inflammatory responses characterized by robust cytokine and chemokine secretion [30]. This inflammatory cascade stimulates reactive oxygen species (ROS) production as a host defense mechanism. However, LPS-induced ROS generation reaches supraphysiological levels, which not only drives macrophage polarization toward the pro-inflammatory M1 phenotype but also suppresses endogenous antioxidant enzyme activity [31,32]. Under the state of oxidative stress induced by diquat, adding tributyrin to the diet could effectively reduce the occurrence of oxidative stress and alleviate intestinal damage by increasing the activity of antioxidant enzymes and reducing the content of ROS [33]. Tributyrin could enhance the antioxidant capacity of the muscle of chickens under stress by activating the Nrf2 signaling pathway, thereby improving the growth performance of chickens and enhancing the meat quality [34]. In the studies on chicken [35], fish [36], and dairy cows [37], tributyrin has shown good antioxidant capacity [38], alleviating stress damage or improving the quality of animal products, and promoting the growth of animals. Our experiment found that dietary supplementation with tributyrin could effectively increase the activities of CAT and GSH-pX antioxidant enzymes in the serum and liver of piglets under LPS-induced stress, reduce the content of MDA, and improve the antioxidant capacity of weaned piglets under LPS-induced stress.

Under stress conditions, the imbalance of the animal’s immune status causes excessive inflammation, resulting in damage to the body and organs [39,40,41]. Our experiment found that intraperitoneal injection of 80 μg/kg LPS into weaned piglets led to a significant increase in AST content in the serum, revealing liver dysfunction and inflammatory injury in the piglets. However, the addition of tributyrin to the diet could significantly reduce the AST content in the serum and effectively alleviate the liver metabolic disorder and injury induced by LPS. Inflammatory response [42,43,44] is a complex immune defense mechanism activated through the synergy of adaptive and innate immunity in the body to resist the invasion of foreign substances [45,46]. Viral and bacterial infections promote M1 polarization of macrophages by activating the NLRP3 inflammasome, leading to robust IL-1β secretion and subsequent amplification of the inflammatory response [47]. Excessive inflammatory responses can generate cytokine storms [48]. The response causes NF-κB p65 to be phosphorylated, enabling it to pass through the cell nucleus and bind to downstream transcription factors. This activates the NF-κB signaling pathway, thereby inducing the expression of inflammatory-related genes/proteins [49,50], and secreting a large amount of inflammatory cytokines [51], such as IL-1β, IL-6, TNF-α, ultimately causing tissue and organ damage or animal death. Interestingly, there exists a dynamic immune response system in animals, which also provides an opportunity for the exogenous addition of new feed additives to regulate the immune imbalance of animals, thereby achieving the effect of preventing and alleviating inflammation. Our study found that liver injury was caused, and a large amount of inflammatory cytokines were produced after LPS induction, while dietary supplementation with tributyrin could reduce the levels of IL-1β and IL-6 in the serum and liver of stress-weaned piglets. And tributyrin significantly reduced the expression levels of *IL-1β*, *IL-6*, and *TNF-α* mRNA, as well as the expression levels of IL-1β precursor and mature proteins. The secretion of inflammatory cytokines was significantly decreased, and the secretion of anti-inflammatory cytokine IL-10 was increased. The above results indicate that dietary supplementation with tributyrin can enhance the antioxidant and immune capabilities of serum and liver in weaned piglets under LPS challenge, and can effectively alleviate liver damage caused by LPS.

Macrophage polarization is associated with inflammatory responses and injury repair, and it is one of the core mechanisms of macrophage function [52]. The NF-κB signaling pathway is a classic signaling pathway that responds to the occurrence of inflammation. When external pathogens invade, the pathogen-associated molecular patterns (PAMPs) are recognized by Toll-like receptors (TLRs), which transmit signals to the cell interior. This process, dependent on MyD88, rapidly activates downstream signaling pathways such as NF-κB and MAPK, promoting the secretion of inflammatory cytokines including TNF-α, IL-6, and IL-1β. Studies have shown that indole-3-butyric acid can effectively alleviate inflammation and arthritis caused by the inflammatory cytokine IL-1β in chondrocytes by regulating the activation of the AHR/NF-κB signaling pathway [53], and regulating NLRP3/NF-κB can effectively improve inflammation and fibrosis caused by non-alcoholic fatty liver disease [54]. The activation of the JAK/STAT signaling pathway can regulate the polarization state of macrophages [55]. Previous studies have found that activating the MVP/JAK/STAT6 signaling pathway of macrophages can promote the transformation of M1-type macrophages into M2-type macrophages [56] and facilitate tissue repair. In another study, total saponins from panax notoginseng promoted M2 polarization in macrophages by regulating and enhancing the phosphorylation of STAT6, thereby alleviating acute lung injury caused by LPS [57]. In this experiment, it was found that dietary supplementation with tributyrin could significantly reduce liver macrophage infiltration induced by LPS and increase the positive rate of M2-type marker CD206. Meanwhile, the results of quantitative real-time PCR showed that tributyrin reduced the mRNA expression levels of M1-type polarization markers *iNOS* and *CD86* in macrophages; promoted the mRNA expression levels of M2-type polarization markers *Arg1*, *IL-4*, and *IL-13*; and significantly increased the mRNA expression levels of *SIRT1*, *JAK2*, and *STAT6*. It was found that tributyrin significantly reduced the relative protein expression levels of p-P65/P65, p-IKBα/IKBα, and SOCS3, as well as significantly increased the protein expression levels of p-JAK2/JAK2 and p-STAT6/STAT6. The above data results indicated that tributyrin can alleviate liver damage caused by LPS by inhibiting the activation of the SIRT1/NF-κB signaling pathway, promoting the activation of the JAK2/STAT6 signaling pathway, inhibiting the secretion of inflammatory cytokines and inflammatory responses induced by LPS, regulating the polarization state of macrophages, and promoting liver tissue repair.

The distinct patterns of interaction between tributyrin and LPS challenge observed across various parameters—from serum enzymes (AST, ALT) and oxidative markers (MDA, CAT) to cytokines (IL-1β, IL-6, IL-10) and macrophage polarization markers (iNOS, CD86, Arg1, F4/80, CD206)—provide crucial insights into the nuanced mechanism of tributyrin’s action. These differential responses can be mechanistically interpreted as tributyrin exerting both preconditioning and context-dependent therapeutic effects. The LPS-independent enhancements, such as the increased JAK2 phosphorylation, suggest a fundamental role of tributyrin in priming key cellular signaling pathways. This baseline elevation in signaling capacity may precondition the animal, creating a metabolic and signaling environment that is more resilient to subsequent inflammatory insults. Conversely, the LPS-dependent effects, where tributyrin’s benefits on pro-inflammatory cytokines (IL-1β, IL-6) and M1 macrophage markers (iNOS, CD86) were evident only under challenge, highlight its targeted therapeutic role. The specificity of this effect is strongly supported by our findings on the SIRT1/NF-κB pathway. Our data reveal that tributyrin significantly increased SIRT1 expression and concurrently reduced the phosphorylation (and thus activation) of NF-κB p65 specifically under LPS challenge. This suggests that the upregulation of SIRT1 is a pivotal mechanism by which tributyrin suppresses the aberrant activation of the NF-κB pathway triggered by LPS [58]. The suppression of this central inflammatory signaling hub logically explains the downstream reduction in the expression of its target genes, including IL-1β, IL-6, and iNOS. Most significantly, the bidirectional polarization of liver macrophages—simultaneous suppression of M1 markers and promotion of M2 markers specifically under LPS challenge—indicates that tributyrin does not merely suppress inflammation but actively reprograms the immune response towards an anti-inflammatory and tissue-reparative state. This shift, potentially mediated through the coordinated modulation of the SIRT1/NF-κB and JAK2/STAT6 pathways as our data suggest [59,60], represents a sophisticated mechanism to resolve inflammation and promote recovery, moving beyond simple anti-inflammatory action to active immune homeostasis restoration [61,62].

## 5. Conclusions

In conclusion, the results of this experiment indicate that dietary supplementation with 0.2% tributyrin increases the average daily gain of weaned piglets during the early post-weaning period. Tributyrin administration promoted the phenotypic transition of liver macrophages from pro-inflammatory M1 to anti-inflammatory M2 polarization in LPS-challenged weaned piglets through modulation of the SIRT1/NF-κB and JAK2/STAT6 signaling pathways. This immunomodulatory effect of tributyrin enhances the antioxidant and immune functions of weaned piglets, ultimately alleviating liver inflammation in weaned piglets under LPS challenge.

## Figures and Tables

**Table 1:** Ingredients and nutrient contents of the basal diet.

Early Phase (0–14 d)		Subsequent Phase (15–28 d)	
Ingredients (%)		Ingredients (%)	
Puffed corn	15.12	Broken rice	10.00
Corn	10.00	Corn	28.79
Flour	25.00	Flour	25.00
Peeled soybean meal	3.00	Peeled soybean meal	18.00
Fermented soybean meal	2.50	Fermented soybean meal	1.50
Puffed soybeans	5.00	Puffed flaxseeds	1.00
Fish meal	5.00	Secondary powder	1.50
Chicken intestinal membrane protein powder	2.50	Chicken intestinal membrane protein powder	1.25
First-grade soybean oil	3.65	First-grade soybean oil	2.00
Glucose	10.00	Glucose	1.25
Feeding biscuit powder	6.00	Feeding biscuit powder	5.00
Premix ^①^	4.00	Premix ^①^	4.00
Acidifying agent	0.30	Acidifying agent	0.30
Salt	0.15	Salt	0.15
Zinc oxide	0.18	Zinc oxide	0.16
Choline chloride (50%)	0.10	Choline chloride (50%)	0.10
Whey powder	5.00	Total	100
Plasma substitute	2.50		
Total	100		
**Nutrient levels**		**Nutrient levels**	
Digestive energy/(MJ/kg)	13.3	Digestive energy/(MJ/kg)	13.05
Crude protein (%)	18.95	Crude protein (%)	17.62
Calcium (%)	0.65	Calcium (%)	0.48
Total phosphorus (%)	0.49	Total phosphorus (%)	0.40
Available phosphorus (%)	0.35	Available phosphorus (%)	0.35
Digestible lysine (%)	1.35	Digestible lysine (%)	1.18
Digestible methionine (%)	0.41	Digestible methionine (%)	0.48
Digestible threonine (%)	0.78	Digestible threonine (%)	0.82

Note: ^①^ Premixes provide for each kilogram of diet: vitamin A 10,000 IU, vitamin D 2000 IU, vitamin E 56 IU, vitamin K 3.0 mg, vitamin B1 4.0 mg, vitamin B6 1.0 mg, vitamin B12 34 ug, vitamin B2 5.0 mg, niacin 32 mg, biotin 0.2 mg, D-pantothenic acid 18 mg, folic acid 3.5 mg, iron 90 mg, copper 90.0 mg, zinc 105 mg, manganese 35 mg, iodine 0.5 mg, selenium 0.2 mg. The units of nutritional components are indicated on the right corner of the table.

**Table 2:** Primer sequences.

Gene	Primer Sequence (5′ → 3′)	Accession Numbers	Size, bp
β-actin	F: CTCCAGAGCGCAAGTACTCC R: AATGCAACTAACAGTCCGCC	XM_003124280.5	153
IL-1β	F: AGCCAGTCTTCATTGTTCAGGT R: CAGGTCATTATTGTTGTCACCGTAG	NM_214055.1	101
TNF-α	F: TTATCGGCCCCCAGAAGGAA R: CGGCTTTGACATTGGCTACAAC	NM_214022.1	129
IL-6	F: AAATGTCGAGGCTGTGCAGA R: TCCACTCGTTCTGTGACTGC	XM_047753916.1	118
IL-4	F: ACACGACGGAGAAGGAAACC R: GTTCCTGTCAAGTCCGCTCA	NM_214123.1	165
IL-10	F: TCGGCCCAGTGAAGAGTTTC R: CGGCATTACGTCTTCCAGGT	NM_214041.1	146
IL-13	F: CTGACCACCAGCATGCAGTA R: CCCGTGGCGAAAAATCATCC	NM_213803.1	219
iNOS	F: ACTGGGTTGAATCTGGGTGAA R: CCAGGGAGTCTGGAGATTTCTTT	NM_001143690.1	164
CD86	F: TGGTGCTGCCTCCTTGAAAA R: GGACACAGACGATGCTCACA	NM_214222.1	593
Arg1	F: TGCTAGACTGCTGAGCAACAT R: CTCCTCGTGGCTGACCC	XM_005659190.2	245
CD206	F: GCCCAGACTGAAGACAGCAT R: GGCATCTACCAGGCAGTTGT	NM_001255969.1	143
SIRT1	F: GAGAAGGAAACAATGGGCCG R: ACCAAACAGAAGGTTATCTCGGT	NM_001145750.2	155
NFκBp65	F: ATGTGGAGATCATTGAGCAGC R: CCTGGTCCTGTGTAGCCATT	NM_001114281.1	151
NFκbiα	F: CAGAATCCCGACCTGGTGTC R: GTCGTCATAGGGCAGCTCAT	NM_001005150.1	231
STAT6	F: AGCCACTACAAACCTGAGCA R: CAGGGGCCATTCCAAGATCA	XM_013997634.2	151
JAK2	F: AGTAGGAGCCGAACCCACA R: TGCCTGCTTCCGAAACCC	NM_214113.1	125

**Table 3:** Information related to antibodies.

Antibodies	Dilution Ratio	Company	Company Information
CD206	1:500	Proteintech	Wuhan, China
F4/80	1:200	Cell signaling technology	Danvers, MA, USA
iNOS	1:1000	Abclonal	Wuhan, China
IL-1β	1:1000	Abcam	Cambridge, MA, USA
SIRT1	1:5000	Proteintech	Wuhan, China
p-P65	1:1000	Affinit biosciences	Jiangsu, China
P65	1:1000	Cell signaling technology	Danvers, MA, USA
p-IKBα	1:1000	Cell signaling technology	Danvers, MA, USA
IKBα	1:5000	Proteintech	Wuhan, China
JAK2	1:1000	Selleck	Houston, TX, USA
p-JAK2	1:1000	Abclonal	Wuhan, China
p-STAT6	1:1000	Affinit biosciences	Jiangsu, China
STAT6	1:1000	Proteintech	Wuhan, China
SOCS3	1:1000	Proteintech	Wuhan, China
β-actin	1:10,000	Proteintech	Wuhan, China

**Table 4:** Effects of tributyrin on the growth performance of weaned piglets.

Items	Group		SEM	*p*-Value
	Control	Tributyrin		
**0–14 d**				
ADG, g/d	318.25 ± 12.96	365.18 ± 14.27 *	15.30	0.045
ADFI, g/d	428.77 ± 12.34	479.46 ± 25.09	14.82	0.087
G:F, g/g	0.74 ± 0.02	0.75 ± 0.02	0.01	0.784
**15–28 d**				
ADG, g/d	530.00 ± 32.30	559.53 ± 20.43	18.75	0.458
ADFI, g/d	829.66 ± 29.64	855.43 ± 31.39	20.95	0.564
G:F, g/g	0.64 ± 0.04	0.66 ± 0.03	0.02	0.757
**0–28 d**				
ADG, g/d	432.19 ± 11.49	450.82 ± 15.60	9.40	0.352
ADFI, g/d	621.60 ± 18.63	648.02 ± 30.29	17.41	0.475
G:F, g/g	0.70 ± 0.01	0.67 ± 0.01	0.01	0.198

Note: Control: fed with a basal diet, tributyrin: fed with a basal diet containing 0.2% tributyrin. Statistical significance was indicated as * *p* < 0.05. ADG: average daily weight gain; ADFI: average daily feed intake; G:F: feed efficiency (gain-to-feed ratio).

## Data Availability

The original contributions presented in this study are included in the article. Further inquiries can be directed to the corresponding author(s).
